# The aspiration test: an arthroscopic sign of lateral meniscus posterior horn instability

**DOI:** 10.1186/s40634-021-00327-0

**Published:** 2021-02-28

**Authors:** Christophe Jacquet, Amanda Magosch, Caroline Mouton, Romain Seil

**Affiliations:** 1 Department of Orthopaedic Surgery, Centre Hospitalier de Luxembourg-Clinique D’Eich, 78 Rue d’Eich, 1460 Luxembourg, Luxembourg; 2 Institute for Movement and Locomotion (IML), Department of Orthopedic Surgery and Traumatology St. Marguerite Hospital, 270 Boulevard Sainte Marguerite, BP 29, 13274 Marseille, France; 3 Luxembourg Institute of Research in Orthopaedics, Sports Medicine and Science, Luxembourg, Luxembourg; 4 Human Motion, Orthopaedics, Sports Medicine and Digital Methods, Luxembourg Institute of Health, Luxembourg, Luxembourg

**Keywords:** Popliteo-meniscal complex, Popliteo-meniscal fascicles, Hypermobile lateral meniscus, Aspiration test, Posterior horn lateral meniscus instability

## Abstract

The suspensory mechanism of the posterior horn of the lateral meniscus (PHLM) is an anatomically complex structure including the popliteomeniscal fascicles, the meniscotibial posterior root attachment and the meniscofemoral ligaments. Damage to one or several of these structures – either through knee trauma or congenital abnormalities—can result in an instability of the PHLM that may lead to lateral knee pain, locking sensations or lack of rotational control of the knee (e.g. after anterior cruciate ligament injuries). The diagnosis of PHLM instability is complex due to the lack of reliable clinical tests and imaging signs. Direct visual dynamic inspection via arthroscopy thus remains the gold standard. However, arthroscopic probing of the PHLM is not always reliable and the precise quantification of the amount of subluxation of the PHLM can be difficult. Therefore, the main objective of this report was to describe a quick and easy arthroscopic screening test called “the aspiration test” in order to help surgeons to detect PHLM instability. During the exploration of the lateral tibiofemoral compartment with the knee kept in the figure of 4 position, the arthroscope is placed in the antero-lateral portal and directed towards the lateral tibiofemoral compartment. The aspiration test is then performed by activating the aspiration of the 4-mm shaver when located in the intercondylar notch. In case of a PHLM instability, an excessive displacement of the PHLM is observed. After repair, a second aspiration test allows to verify that the PHLM has been stabilized.

**Supplementary Information:**

The online version contains supplementary material available at 10.1186/s40634-021-00327-0.

## Introduction

Identifying an instability of the posterior horn of the lateral meniscus (PHLM) can be challenging due to the lack of an appropriate, dynamic method to confirm the diagnosis. Instability of the PHLM can result from a traumatic or an atraumatic insufficiency of the posterolateral suspensory complex which includes the popliteomeniscal fascicles [24], the meniscotibial posterior root attachment and the meniscofemoral ligaments [9]. It may induce locking sensations during deep knee flexion and can be isolated or observed in association with anterior cruciate ligament (ACL) or posterolateral corner injuries [3, 6, 27, 28]. Recently, it has been shown that instability of the PHLM may impact knee rotational instability [17, 25, 27]. It is therefore crucial to further investigate this entity to improve its diagnosis and to allow for complete anatomical repair when indicated.

Several structures of the posterolateral suspensory complex contribute to the active and passive stability of the knee: the popliteus tendon and the popliteo-meniscal fascicles (PMF) [12, 16, 32], the meniscofemoral ligaments (Humphrey and Wrisberg) [9, 13, 28] and the posterior root of the lateral meniscus [9, 25]. Injuries to the posterolateral suspensory complex of the LMPH are commonly under-recognized due to the lack of consistent clinical or MRI findings [14, 15, 26]. In a study by Simonian et al. [26], none of the patients with a instability of the PHLM at the time of surgery presented abnormal preoperative MRI findings. As MRI assesses the knee in static conditions, it may indeed not allow diagnosing the instability of the PHLM that occurs during knee motion.

An arthroscopic confirmation of the instability of the PHLM remains the diagnostic gold standard. Shin et al. [24] suggested that the instability was confirmed when more than half of the lateral meniscus could be extruded during arthroscopic probing. This led to define the lateral meniscus as being hypermobile. But arthroscopic probing may not always be a reliable test to diagnose the instability of the PHLM. There is thus a need to improve the detection of this condition during arthroscopic exploration. The main objective of this report was to describe an arthroscopic screening test called “the aspiration test” to help surgeons to better detect the instability of the PHLM.

## Techniques for arthroscopic evaluation of the PHLM

The diagnosis of the instability of the PHLM cannot be made by a single test. It is the sum of several individual clinical signs (patient history, clinical examination, imaging, arthroscopic findings). A reliable diagnosis of PHLM instability would ideally require a systematic visual dynamic inspection of the lateral meniscus under arthroscopy, currently considered as the gold standard. Direct arthroscopic inspection of the posterolateral suspensory complex of the PHLM including popliteus tendon, meniscofemoral ligaments and the PMF’s as well as the interpretation of the arthroscopic findings is however challenging. Currently, there are 2 arthroscopic methods to evaluate the stability of the PHLM. Both offer complementary information.

### The lateral drive through sign

**Fig. 1:**
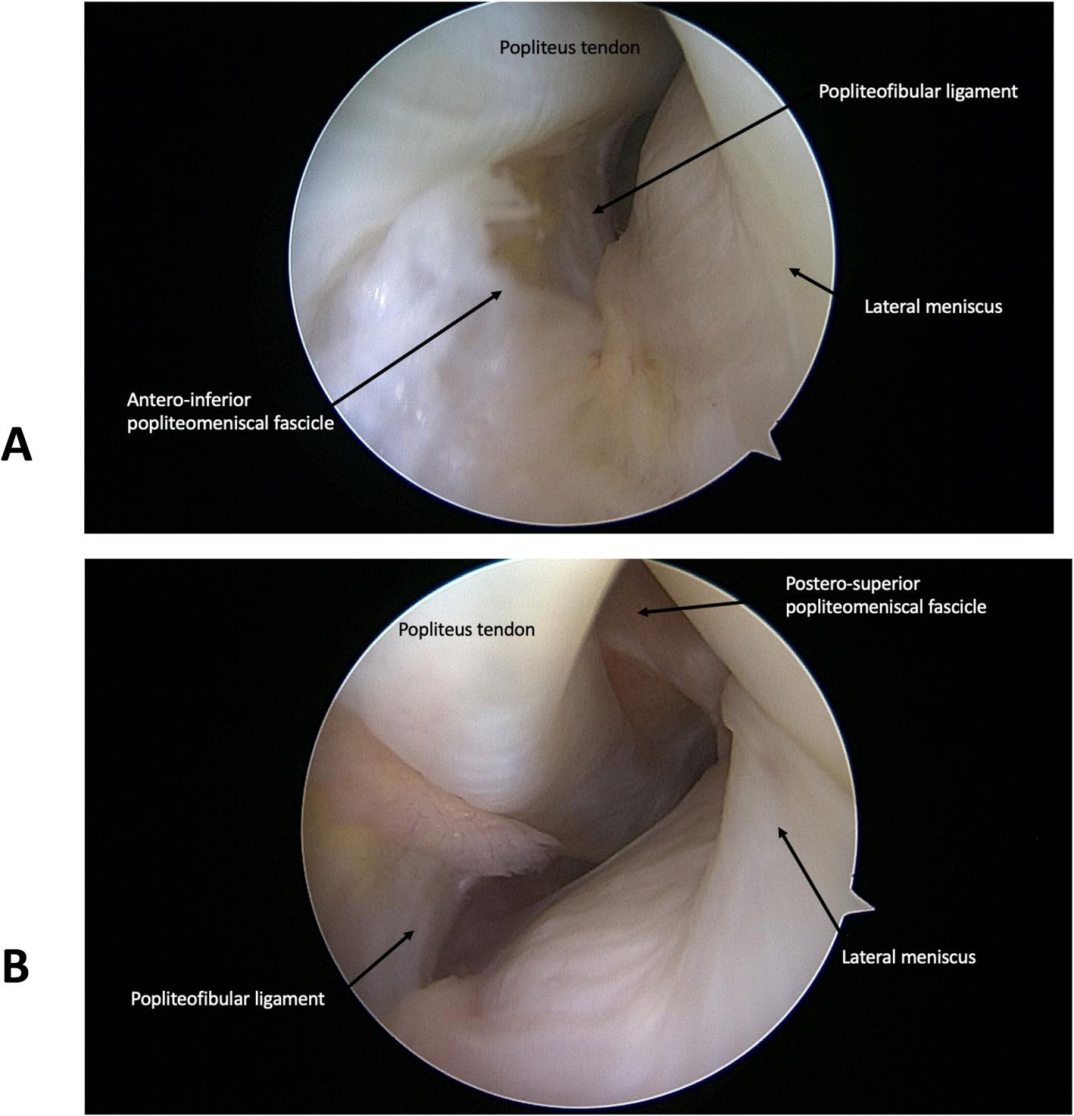
The lateral drive through sign showing the popliteus tendon, the antero-inferior popliteomeniscal fascile (**a**) and the postero-inferior popliteomeniscal fascicle (**b**)

The lateral drive through sign is performed in the extended knee. The arthroscope is advanced distally and caudally in the lateral gutter until visualization of the popliteus hiatus and the popliteus tendon. Knee flexion is then increased to 90° to ‘plunge’ the arthroscope in the popliteal space. This technique allows the visualization of the posterior tibia, the menisco-tibial capsular attachments, the popliteo-fibular ligament, the postero-superior PMF (PS-PMF) (Fig. 1b), the posterior lateral femoral condyle and the posterior aspect of the lateral meniscus. The antero-inferior PMF (AI-PMF) may sometimes be visible as well (Fig. 1a). The popliteus tendon is visible at its midportion only, as the femoral attachment site and its musculotendinous junction are difficult to visualize [7].

This visualization technique is challenging and may not be easy to perform for beginners. Beneath the technical difficulties related to the direct visualization of the posterolateral complex, interpreting the findings is highly related to the surgeon's skills and has limited reproducibility because of a lack of standardization and scientific evaluation.

### Anterior arthroscopic inspection of the PHLM

**Fig. 2:**
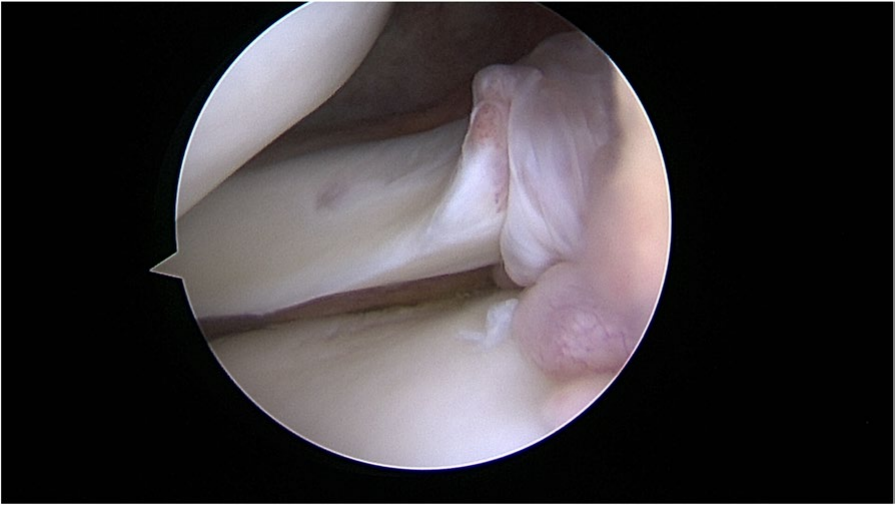
Incomplete root tears with an elongation of its meniscotibial attachment fibers

The second arthroscopic method to evaluate the stability of the PHLM is the classic anterior view with the knee held in a figure-of-4 position [16] This method has several disadvantages like the impossibility or difficulty to directly assess the PMF’s and the limited joint line opening of the lateral tiobiofemoral compartment in narrow knees. But it however does allow for a direct visualization of the posterior root of the lateral meniscus (PRLM) and of the meniscofemoral ligaments. The diagnosis of PLRM injuries is commonly based on direct visualization of the tear or by the avulsion of the root during probing [4, 6]. In case of an incomplete root tear with an elongation of its meniscotibial attachment fibers (Video 1 and Fig. 2), the probing test is sometimes insufficient to unmask the instability of the PHLM. In case of an excessive mobility of the lateral meniscus during probing, the diagnosis of the PHLM instability can be made. However, this method is not always reliable and does not allow to precisely quantify the amount of subluxation of the PHLM.

### The aspiration test

The authors noticed a frequent discrepancy between instability of the PHLM at probing and with aspiration (Video 1). We therefore propose a quick and easy screening test to evaluate the instability of the PHLM: the aspiration test. At the time of the exploration of the lateral tibiofemoral compartment with the knee held in the figure of 4 position [16] and flexed to slightly more than 90°. The arthroscope is placed in the antero-lateral or antero-medial portal and directed towards the lateral tibiofemoral compartment. The aspiration test is easily performed by completely activating the aspiration of a 4,5 mm shaver placed at the center of the lateral tibiofemoral compartment (arthroscopy pump with standard knee configuration: DualWawe, Arthrex, Naples, FL, USA). The test can be repeated to allow for an ideal placement of the arthroscope or the shaver. As aspiration can trigger bleeding, it should then be performed during a shorter amount of time. Despite bleeding, this test has no known risk of structural meniscus injuries. In the absence of a pathological instability of the PHLM, no anterior translation is observed. Conversely, the diagnosis of instability of the PHLM can be confirmed in the presence of an excessive translation of the most unstable part of the posterior portion of the lateral meniscus to, or close to, the center of the lateral tibial plateau with fluid aspiration (Video 2). The location of the displaced portion of the PHLM may indicate whether the instability is merely caused by an insufficiency of the AI-PMF, the PS-PMF (Video 2 and Video 3), the root attachment area or by a combination of insufficiencies in these structures. To obtain a reliable information during the aspiration test, care should be taken not to immobilize one of these areas with the shaver or the camera.

In case of an excessive anterior translation of the PHLM during the aspiration test, an additional evaluation by probing the PHLM and attempting to actively displace it into the joint can be performed. According to Shin et al., a translation of the lateral meniscus by more than 50% or 'beyond the equator' of the lateral femoral condyle, is considered as hypermobility [24]. This mobility may be influenced by the force exerted with the probe as well as the degree of opening of the lateral femoro-tibial compartment. It thus appears to be poorly reproducible. After repair of the PHLM and its suspensory mechanism, a second aspiration test allows to verify that the PHLM has been stabilized. (Video 1 and Video 4).

## Discussion

The most important information of this report was the description of the aspiration test, an easy and quick arthroscopic test to detect an instability of the PHLM. The description of this test is important because the diagnosis of PHLM instability is often delayed or missed due to unclear and inconsistent clinical and imaging findings with no evident meniscal or articular cartilage abnormalities. Likewise, structural damage of the suspensory mechanism of the PHLM consisting of the popliteomeniscal fascicles, the meniscotibial posterior root attachment and the meniscofemoral ligaments is rarely obvious, even under direct arthroscopic visualization. The aspiration test is therefore useful to firmly identify the instability of the PHLM as well as to verify its stabilization after arthroscopic repair.

The anatomy of the suspensory mechanism of the PHLM is complex and its structural tissue damage difficult to evaluate arthroscopically. The PMF's are posterolateral menisco-capsular extensions that blend inferiorly into the musculotendinous portion of the popliteus. They allow the tendon to pass from an intraarticular to an extraarticular location while maintaining the compartmental integrity of the knee joint. At the height of the popliteal hiatus, the popliteus tendon attaches to the PHLM via at least two PMF: an anteroinferior (AI)-PMF and a posterosuperior (PS)-PMF [22]. Altogether, these fascicles form the hoop-like appearance of the popliteal hiatus. The PHLM has also two variable fibrous attachments from the femur running in front of and behind the PCL, the anterior meniscofemoral ligament (Humphrey), and the posterior meniscofemoral ligament (Wrisberg) [28]. Finally, the meniscus root which has been defined by Brody et al. [5] ‘‘as the last few millimeters of meniscal tissue angling down to the tibial plateau attachment in the intercondylar notch”.

The pathophysiology of PHLM instability remains controversial. A frequent origin is a congenital deficiency of the peripheral attachments, like the Wrisberg-variant type of the discoid meniscus [10, 19] where the meniscotibial ligament or root attachment is absent, but the PHLM presenting an otherwise near normal shape [21, 33]. Another cause is that PHLM instability may occur in traumatic conditions, such as in ACL injuries. In these, the instability may be caused by a subtle and often invisible structural damage to the suspensory mechanism of the PHLM. ACL injuries typically occur during a combined anterior translation and external rotation of the tibia against the femur [8] causing a blow of the posterolateral tibial plateau against the lateral femoral condyle typically resulting in a bone bruise or an impression [11]. At the moment of anterior subluxation, the posterolateral suspensory complex of the PHLM is squeezed and massively strained between the femur and the tibia. The exerted shear forces may lead to a lateral meniscus tear or a structural damage to the suspensory mechanism of the PHLM. The incidence of these lesions however currently remains unknown. The high prevalence of lateral meniscus root tears (17% [18]) and lateral femoral and tibial bone bruises/impression fractures in association with ACL tears however suggests that a significant number of tears of the suspensory mechanism of the PHLM remains undiagnosed and untreated.

Several studies attempted to evaluate the structure and functions of the PMFs and its relations to the lateral meniscus and knee stability. In a cadaveric study, Simonian et al. [26] demonstrated an 78% average increase in anterior knee displacement at 90° of knee flexion with a 10-N load after cutting both the AI and PS-PMF. In their recent investigation comparing MRI and arthroscopic findings, Suganuma et al. [30] demonstrated the clinical importance of these structures by analyzing their presence or absence in recurrent subluxations of the lateral meniscus (RSLM) in stable knees. Abnormal PS-PMFs and AI-PMFs were found in 40 and 26%, respectively, in a control group of 215 healthy knees. An abnormal PS-PMF was identified in 100% of the knees with RSLM (*n* = 16) and 100% of the contralateral knees of patients with RSLM (*n* = 7). Abnormal AI-PMF was found in 100% of knees with RSLM compared to only 29% in the contralateral knees of patients with RSLM. An abnormal AI-PMF therefore seems to be the source of symptomatic instability of the lateral meniscus.

The biomechanical consequences of a PHLM root avulsion or elongation and meniscofemoral ligament injuries on PHLM stability have received little attention so far. The simulation of a complete radial root tear of the PHLM in a finite element model could however show that the lateral meniscus was only slightly displaced in a radial pattern by a compressive load. Additional insufficiency of the posterior meniscofemoral ligament, however, markedly increased the amount of the meniscal displacement [2]. Another study by Simonian et al. [26] confirmed that the section of PFMs increased significantly meniscal motion but did not determined meniscal displacement in the notch.

Despite the fact that the instability of the PHLM is directly associated with the lateral meniscus, distinct symptoms consistent with lateral meniscus pathology are uncommon. Knee locking in deep flexion however seems to be one of the repeating complaints in isolated PHLM instabilities [32]. In the absence of clear meniscal symptoms and imaging findings, mechanical symptoms should thus raise suspicion of a PHLM instability as part of the differential diagnosis [1].

The knowledge about the MRI appearance of the suspensory complex of the PHLM is still insufficient. It is known from dissection study that the anatomy may vary from one subject to another. The presence or absence of PMF has been recorded with some variance [23]. Furthermore, Tria et al. [31] reported that 18 of 40 cadaveric knees had an isolated insertion of the popliteus tendon to the lateral femoral condyle with no connection to the lateral meniscus. Munshi et al. [20] reported seven of seven cadaveric knees with two fasciculi, also detectable in corresponding MRI images. Suganuma et al. [30], also recently reported on the use of magnetic resonance imaging (MRI) in the diagnosis of PMF tears. MRI however assesses the knee in static conditions and may not allow diagnosing the instability of the LMPH that occurs during knee motion as confirmed by Simonian et al. [26] who highlighted that patients with an unstable PMF tears at the time of surgery had normal MRI results. Similarly, Krych et al. [14] observed that on 45 patients with arthroscopically confirmed PHLM tears, only 15 (33%) were initially diagnosed on preoperative MRI. Arthroscopy and visual dynamic inspection of the lateral meniscus thus remains the gold standard in the diagnosis of PHLM instability.

The aspiration test has some advantages compared to the probing test. The traction force exerted during the aspiration test is standardized and operator-independent. Likewise, the aspiration force is equally applied to the entire structure of the PHLM and not only to the extremity of the arthroscopy probe. An alternative to the aspiration test has been described in the study by Steinbacher et al. [29] and was called the Tom’s test in which an aspiration force is generated through the arthroscope and not the shaver. It has the disadvantage to negatively influence the visibility of the PHLM.

There are several limitations to the present report. The goal was not to evaluate the sensitivity or specificity of this test in the absence of a diagnostic gold standard. In the authors’ current surgical practice, the presence of clinical symptoms suggestive of PHLM instability associated with a positive aspiration test systematically lead to a repair to stabilize the PHLM by using an all-inside repair technique. Another limitation is that this study did not look into the correlation with MRI, but it seems unsuitable for the diagnosis of this pathology. Further investigations are needed to evaluate the presence of a positive aspiration test in normal knees without PHLM instability symptoms as well as in patients with ACL injury.

## Conclusion

The aspiration test is a quick and easy arthroscopic test which can be performed in daily practice to evaluate the instability of the PHLM.

## Supplementary Information

**Additional file 1: Video 1**. Right knee with the 30° arthroscope in the antero-lateral portal. Incomplete root tears with an elongation of its meniscotibial attachment fibers with a discrepancy between instability at probing and with aspiration and a negative aspiration test after reparation.

**Additional file 2: Video 2**. Left knee with the 30° arthroscope in the antero-lateral portal. Positive aspiration test with displacement of the posterior part of the PHLM reflecting an insufficiency of the PS-PMF.

**Additional file 3: Video 3**. Left knee with the 30° arthroscope in the antero-medial portal. Positive aspiration test with displacement of the posterior and the anterior part of the PHLM reflecting an insufficiency of the PS-PMF and AI-PMF.

**Additional file 4: Video 4**. Left knee with the 30° arthroscope in the antero-medial portal. Negative aspiration test after reparation of the case presented in the video 2.

## Data Availability

Not applicable.
